# Attosecond streaking measurement of extreme ultraviolet pulses using a long-wavelength electric field

**DOI:** 10.1038/srep35594

**Published:** 2016-10-18

**Authors:** Nariyuki Saito, Nobuhisa Ishii, Teruto Kanai, Shuntaro Watanabe, Jiro Itatani

**Affiliations:** 1 The Institute for Solid State Physics, The University of Tokyo, Kashiwanoha 5-1-5, Kashiwa, Chiba 277-8581, Japan; 2 Research Institute for Science and Technology, Tokyo University of Science, Yamasaki 2641, Noda, Chiba 278-8510, Japan

## Abstract

Long-wavelength lasers have great potential to become a new-generation drive laser for tabletop coherent light sources in the soft X-ray region. Because of the significantly low conversion efficiency from a long-wavelength light field to high-order harmonics, their pulse characterization has been carried out by measuring the carrier-envelope phase and/or spatial dependences of high harmonic spectra. However, these photon detection schemes, in general, have difficulty in obtaining information on the spectral phases, which is crucial to determine the temporal structures of high-order harmonics. Here, we report the first attosecond streaking measurement of high harmonics generated by few-cycle optical pulses at 1.7 μm from a BiB_3_O_6_–based optical parametric chirped-pulse amplifier. This is also the first demonstration of time-resolved photoelectron spectroscopy using high harmonics from a long-wavelength drive laser other than Ti:sapphire lasers, which paves the way towards ultrafast soft X-ray photoelectron spectroscopy.

Progress in carrier-envelope phase (CEP)-controlled intense femtosecond lasers has opened the path to the generation of attosecond pulses in the extreme ultraviolet (XUV) region via high harmonic generation (HHG). Such ultrashort XUV pulses have been applied to attosecond spectroscopy, where photo-induced electronic dynamics are probed on unprecedented time scales^1^. Characterization of attosecond XUV pulses is vital because it is the basis of attosecond measurements. So far, various characterization techniques of attosecond pulses such as nonlinear auto-correlation measurement^2^,^3^, reconstruction of attosecond beating by interference of two-photon transitions (RABBITT)^4^ and attosecond streaking^5^,^6^,^7^ have been proposed and demonstrated. Especially, RABBITT and attosecond streaking techniques are both based on time-resolved photoelectron spectroscopy using two-colour electric fields; one is a high-frequency field that induces single-photon ionization, and the other is an intense low-frequency field that modifies the photoelectron spectra in a phase-sensitive manner. These two techniques are proven very useful for tracing attosecond electron dynamics in atoms^8^,^9^ and solids^10^. However, these experiments have been conducted dominantly with Ti:sapphire-based light sources operating around 800 nm.

Recently, long-wavelength light sources based on optical parametric amplifiers (OPAs) or optical parametric chirped-pulse amplifiers (OPCPAs) have been developed^11^,^12^,^13^,^14^,^15^,^16^ and applied to HHG to extend the cutoff energy towards the soft X-ray region, where Ti:sapphire-based sources cannot access. Using high-energy low-repetition-rate IR or mid-IR sources, high harmonic cutoff has reached the water window^17^ and even beyond 1 keV^18^. CEP-dependent soft X-ray spectra beyond the carbon *K-*edge have also been observed using high-repetition-rate, CEP-stable IR sources, indicating the generation of isolated soft X-ray bursts^19^,^20^.

As for the pulse characterization of high harmonics generated by long-wavelength sources, several methods based on *photon* detection have been reported^20^,^21^,^22^. The advantage of these methods is that they do not require high photon flux, thus compensate the low conversion efficiency of HHG which scales with *λ*^−(5–6) ^23,24,25. Yet, the major drawback is that they cannot directly access the information of temporal phases or they require strong assumptions regarding the HHG process. Therefore, *photoelectron*-based characterization techniques such as attosecond streaking are preferable because they can directly access full temporal information of high harmonics. However, angle-resolved photoelectron measurements require a high photon flux to obtain reasonable electron signals, which is a big challenge in attosecond science with long-wavelength sources.

In this work, we realize attosecond streaking measurements using high harmonics driven by a long-wavelength field for the first time. Using a high-energy IR source and optimizing the HHG scheme, we obtain a high photon flux around 100 eV, which is sufficient for photoelectron measurements. This is an important step for realizing ultrafast XUV and soft X-ray spectroscopy with long-wavelength IR sources.

## Experiment

**Figure 1:**
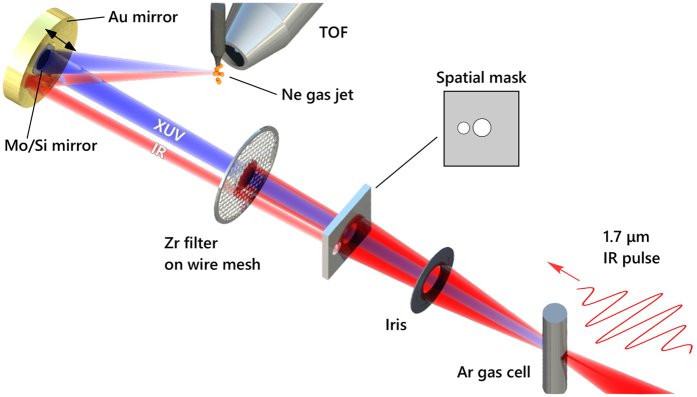
Schematic of the attosecond streaking measurement. 1.7 μm IR pulses were loosely focused into an argon gas cell to generate XUV high harmonics. The XUV and IR beams propagated collinearly, then the two beams were separated by a spatial mask and a filter module. The spatial mask consisted of an aluminium plate with two adjacent holes. The filter module had a zirconium filter at the center which transmitted the XUV beam alone. The outer part of the IR beam transmitted through a wire mesh that supported the zirconium filter. The separated beams were focused into a neon gas jet by a double concave mirror module which consisted of a gold mirror for the IR beam and a Mo/Si mirror on a piezo delay stage for the XUV beam. The intensity of the IR pulse at the gas jet was adjusted by an iris before the spatial mask. Photoelectrons from neon were measured by a time-of-flight(TOF) spectrometer.

Figure 1 shows a schematic of our attosecond streaking beamline. Output pulses from our high-energy BiB_3_O_6_ (BIBO)-based OPCPA (pulse energy: 1.5 mJ, central wavelength: 1.7 μm, repetition rate: 1 kHz)^26^ were loosely focused (*f* = 1 m) into a 15-mm-thick argon gas cell with a backing pressure of 50 mbar. The focus spot size was measured to be around 300 μm (at 1/*e*^2^ intensity). This loose focusing condition resulted in the reduction of the phase mismatch and the increase of the interaction length in HHG. The energy of the IR beam was adjusted to 570 μJ by an iris in front of a focusing mirror to set the cutoff energy slightly below 100 eV, near the silicon *L-*edge. From this cutoff energy, the peak intensity of the IR pulses was estimated to be ~9.2 × 10^13^ W/cm^2^. This value is consistent with an independently obtained IR intensity of 1.2 × 10^14^ W/cm^2^ from a measured IR pulse energy of 570 μJ, a spot diameter of 300 μm and a pulse duration of 12.7 fs determined from the streaking measurement. At 1-m downstream after the argon gas cell, we introduced a spatial mask and a filter module to separate the XUV radiation from the IR beam. The spatial mask was an aluminium plate with two adjacent holes (on-axis: 6 mm in diameter, off-axis: 4 mm in diameter). The filter module consisted of a zirconium filter (150 nm thick, 4 mm in diameter) mounted on a nickel wire mesh, where the on-axis IR beam was blocked and the off-axis IR beam was transmitted through. The on-axis XUV beam with a measured divergence of 3.8 mrad (full width at 1/*e*^2^) was focused into a neon gas jet by a concave Mo/Si multilayer mirror, which had a 5-eV bandwidth below the silicon *L*-edge with a focal length of 300 mm. The incident angle of the Mo/Si mirror was about one degree to avoid the spatial overlap of the incoming and reflected beams at the gas jet while minimizing the astigmatism. The off-axis IR beam was also focused into the neon gas jet by a gold concave mirror, which had the same focal length as the Mo/Si mirror. The intensity of the IR beam at the focus was adjusted by an iris before the spatial mask. The IR and XUV beams non-collinearly intersected with an angle of less than two degrees, which did not significantly affect the attosecond streaking^27^. The neon gas flow was supplied from a 15-μm-inner-diameter glass capillary at a rate of 4.9 sccm and a backing pressure of 2.8 bars. In this setup, the target gas was estimated to absorb 0.1% of the XUV photons, 0.88% of which were detected by a micro-channel plate (quantum efficiency: 70%) in a time-of-flight electron spectrometer assuming cos^2 ^*θ*-dependence of photoelectron emission. A typical count rate of XUV photoelectrons was about 20 counts per second (0.02 counts per shot). From the count rate and the detection efficiency of photoelectrons, the XUV flux is estimated to be 3200 photons/pulse (49 fJ/pulse) at the streaking. The acquisition time for one spectrum was 45 seconds and the total acquisition time was about two hours. The relative delay between the IR and XUV pulses was controlled by translating the Mo/Si mirror on a piezo-controlled delay stage with respect to the outer gold mirror. The electron spectra were collected with delay steps of 40 nm, corresponding to 267 attoseconds. The CEP was carefully controlled by looking at the streaked electron spectrum at the maximum of the vector potential of the IR field. If there is a satellite pulse next to the main attosecond pulse, the electron wavepackets released by these two pulses are streaked in the opposite directions. By minimizing the satellite component, we chose a CEP to generate isolated attosecond pulses.

## Results

**Figure 2:**
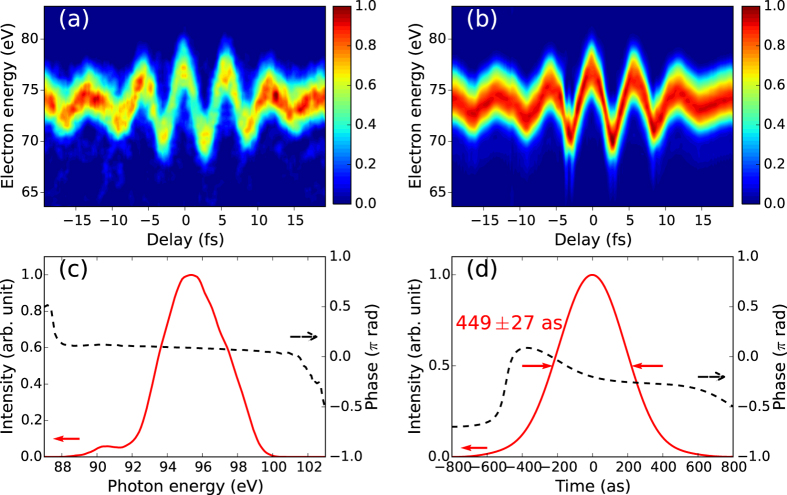
Results of attosecond streaking pulse characterization. (**a**) Measured and (**b**) reconstructed streaking spectrograms. A positive delay corresponds to the XUV pulse arriving after the IR pulse. (**c**) Retrieved spectrum and (**d**) temporal profile of the XUV pulse. The measured pulse duration was 449 ± 27 as FWHM.

**Figure 3:**
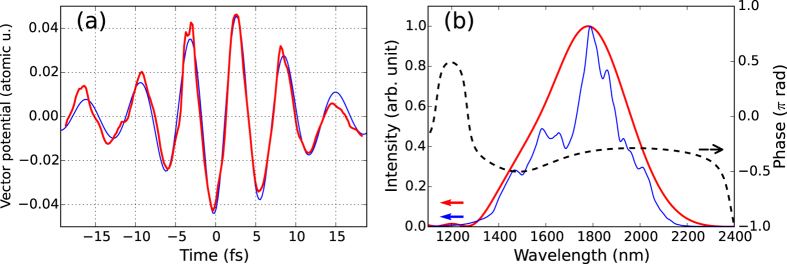
IR pulse properties retrieved from the streaking measurement. (**a**) Waveform of the vector potential which was retrieved from the streaking spectrogram (red line) and from the spectrum measured by a grating spectrometer assuming a flat phase (blue line). (**b**) Spectral intensity and phase of the IR electric field. The red and blue lines represent spectral intensities retrieved from the streaking and measured by the grating spectrometer, respectively. In the short wavelength region below the plot range, the red line has several peaks due to noises and harmonics, which are not shown here. The black dashed line shows the spectral phase retrieved from the streaking.

Figure 2(a) shows the measured streaking spectrogram. Field-induced modulation in the photoelectron spectra is clearly observed, which implies the generation of isolated sub-femtosecond XUV bursts. The FROG-CRAB (PCGPA) algorithm^28^ is used to reconstruct the measured spectrogram as in Fig. 2(b) and to retrieve the XUV pulse. The retrieved XUV spectrum and the temporal profile are plotted in Fig. 2(c,d), respectively. The duration of the retrieved XUV pulse is 449 ± 27 attoseconds. The error is calculated from the final signal matrix of the algorithm^29^. The retrieved pulse duration reaches the transform-limited value of 441 attoseconds within the experimental error. The nearly attochirp-free pulse shape is attributed to the fact that the XUV spectrum is close to the cutoff energy. A previous study using an IR source showed that the attochirp was positive in the plateau region because of the large contribution of short trajectories^22^. We also observe positive chirp in attosecond pulses that are produced by IR pulses at slightly higher intensity. This observation implies an extension of the cutoff and increased contribution of the short trajectories. We also retrieve the vector potential of the streaking IR pulse by FROG-CRAB. Its waveform and spectrum are plotted in Fig. 3 (red line and black dashed line). The duration of the retrieved IR pulse is 12.7 fs FWHM and the peak intensity is 4.6 × 10^10^ W/cm^2^. For comparison, the spectral intensity measured by a grating spectrometer and a vector potential calculated from it assuming a flat phase are also plotted in Fig. 3 (blue line). The good agreement between the red and blue lines in Fig. 3(b) supports the reliability of the streaking measurement.

## Conclusion

We generate XUV pulses below the Si *L*-edge from argon gas irradiated by few-cycle, 1.7-μm pulses from the BIBO-based OPCPA. The generated XUV pulses are characterized using the attosecond streaking technique with the IR electric field, proving the applicability of the long-wavelength OPCPA source for attosecond streaking. Extension of the IR-field-driven attosecond streaking from XUV to soft X-ray region is still a challenge due to lower photon flux. However, based on the obtained results, attosecond streaking at the carbon *K*-edge is feasible by: (i) optimizing the HHG process using a high-pressure gas cell, which was recently demonstrated^18^,^30^, (ii) changing the target gas from neon to argon to increase the soft X-ray absorption using the inner shell excitation, and (iii) improving the collection efficiency of the photoelectron spectrometer.

## Methods

### Long-wavelength light source

We used a millijoule BIBO-based OPCPA system for the attosecond streaking, which has been previously described elsewhere^26^. Here, we present minor modifications to the OPCPA system for the streaking measurements. As in the ref. 26, we previously utilized gain saturation and the red shift in a Ti:sapphire amplifier for the generation of pump pulses used in BIBO OPA stages to strongly amplify the blue components of an IR seed spectrum. The blue parts of the IR seed were concentrated in time, because the third-order dispersion centred at 1270 nm was introduced during the pulse stretching of the IR seed pulses, which were compressed in a 150-mm-long synthetic fused silica block after the BIBO OPA stages. This scheme, however, turned out to be sensitive to the timing-jitter between the pump and seed pulses in the OPAs, resulting in the fluctuation of photoelectron counts, which was around 30% rms for 1.5 hours with an acquisition time of 20 seconds. Hence, we modified a spatial mask, which was previously used to select a 20-nm-wide, near-rectangular spectrum centred at 805 nm, to obtain spectrally flat pump pulses. Accordingly, the red components of the IR seed pulses are amplified more than the blue components, which results in the shift of the central wavelength from 1.6 μm to 1.7 μm. After this modification, the photoelectron fluctuation was reduced to 15% rms. The energies of the pump and the amplified IR pulses are similar to those described^26^.

### CEP dependence of the XUV spectra

**Figure 4:**
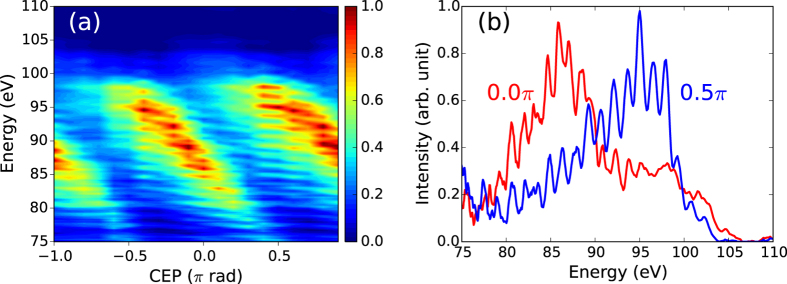
CEP-dependent HHG near the silicon *L-*edge. (**a**) CEP-dependent XUV spectra with steps of 0.1π rad, clearly showing transition between an interference pattern and continuum near the cutoff. (**b**) XUV spectra obtained at a CEP of 0.0 π rad (red line) and 0.5 π rad (blue line) in the CEP scan respectively, indicating the isolated XUV pulses with a continuum bandwidth of ~10 eV above 95 eV at 0.0 π rad.

We conducted an HHG experiment in a different beamline using the same IR source. The beamline used for the HHG experiment is described elsewhere^19^. Briefly, the IR beam with a diameter of 7 mm was focused (*f* = 500 mm, f/71) into a 12-mm-thick argon gas cell with a backing pressure of 50 mbar. The IR energy was adjusted to obtain a high harmonic cutoff energy of around 100 eV. The fundamental beam and harmonics below 60 eV were blocked by a 150-nm-thick zirconium filter. The transmitted harmonics were measured by an XUV spectrometer (SXR-II-1, Hettrick Scientific) with an X-ray CCD camera (PIXIS-XO-2048B, Princeton Instruments). The exposure time of the camera was set at three seconds. Figure 4(a) shows the CEP dependence of the XUV spectra obtained with steps of 0.1 π rad. The cutoff energy of the harmonics reached 105 eV. The peak laser intensity was estimated to be about 9.5 × 10^13^ W/cm^2^ from the strong-field approximation-based simulation. The red and blue lines in Fig. 4(b) show XUV radiation spectra at relative CEP values of 0.0 π rad and 0.5 π rad, respectively. The red line clearly shows two half-cycle cutoffs located at around 95 eV and 105 eV. A continuum spectrum can be seen between them, indicating the generation of an isolated XUV burst. Below 95 eV, a comb-like structure can be seen, which originates from interference between consecutive XUV bursts.

## Additional Information

**How to cite this article**: Saito, N. *et al*. Attosecond streaking measurement of extreme ultraviolet pulses using a long-wavelength electric field. *Sci. Rep.* **6**, 35594; doi: 10.1038/srep35594 (2016).
